# ‘I am tired, sad and kind’: self-evaluation and symptoms of depression in adolescents

**DOI:** 10.1186/s13034-023-00661-4

**Published:** 2023-11-08

**Authors:** Emily Hards, Faith Orchard, Shirley Reynolds

**Affiliations:** 1 Department of Psychology, University of Bath, Claverton Down, Bath, BA2 7AY UK; 2 School of Psychology, University of Sussex, Sussex, UK; 3 School of Psychology and Clinical Language Sciences, University of Reading, Earley Gate, Whiteknights Road, Reading, RG6 7BE UK

**Keywords:** Adolescents, Cognitive theory, Depression, Self-concept, Self-evaluation, Twenty statements test

## Abstract

**Introduction:**

Although self-evaluation i.e., negative perceptions of the self is a common depression symptom in adolescents, little is known about how this population spontaneously describe their self and available data on adolescent self-evaluation is limited. This study aimed to generate and report on a list of words used by healthy adolescents and those with elevated depression symptoms to describe their self-evaluation. Linguistic analysis (LIWC) was then used to compare self-evaluation between the two groups.

**Methods:**

Adolescents aged 13–18 years (n = 549) completed a measure of depression symptoms (the Mood and Feelings Questionnaire) and a measure of self-evaluation (the Twenty Statements Test). Responses were then collated and presented in a freely accessible resource and coded using Linguistic Inquiry Word Count (LIWC) analysis.

**Results:**

Self-evaluation words generated by adolescents were uploaded to a publicly accessible site for future research: 10.15125/BATH-01234. Adolescents with elevated depression symptoms described themselves as ‘Tired’ and ‘Sad’ more than healthy adolescents. However, there was no difference between groups in respect to their use of specific positive, prosocial self-evaluation ‘words’ (i.e., ‘Caring’ and ‘Kind). Following Linguistic Inquiry Word Count (LIWC) analysis, adolescents with elevated depression symptoms generated significantly more words than healthy adolescents, generated more words classified as negative emotion, anxiety and sadness and generated fewer words classified positive emotion than healthy adolescents.

**Conclusions:**

As predicted by the cognitive model of depression, our findings suggest that adolescents with elevated symptoms of depression generated more negative self-evaluation words than healthy adolescents; however they also generated prosocial positive self-evaluation words at the same rate as non-depressed adolescents. These novel data therefore identify an ‘island’ of resilience that could be targeted and amplified by psychological treatments for adolescent depression, and thus provide an additional technique of change.

## Background

Adolescence has been identified as an important period for the development of self-concept [1]. Developing advanced cognitive abilities such as abstract thinking, enables young people to construct more complex representations of who they are, i.e. their ‘self-concept’[2, 3] and to hold complex mental images of themselves that include both positive and negative (e.g. “I am kind”; “I am ugly’” Hards, Fisk, Ellis & Reynolds, 2019). The development of the self-concept interacts with observable changes in mental health and well-being, including difficulties that frequently emerge during adolescence, e.g., major depression and social anxiety. For example, negative self-evaluation is one of the most frequently reported symptoms of depression amongst young people [4].

Depressive disorders, as well as sub-threshold symptoms of depression, are common during adolescence [5, 6]. A diagnosis of major depression during adolescence is associated with a range of immediate and long-term adverse consequences including an increased risk of suicidal thoughts and behaviours [7], difficulties in education and employment [8], and relationships [9]. The cognitive model of depression [10] suggests that pervasive and sustained negative beliefs about the self, the world and the future (i.e. the ‘cognitive triad’) increase vulnerability to depression and after the onset of depression maintain low mood. Evidence-based treatments for depression in young people are moderately successful [11, 12] but often fail to engage young people. These treatments are adapted from treatments developed for adults and thus may not integrate or tackle aspects depression that are characteristic of adolescents and which therefore are more salient and relevant to them, and more effective.

Negative self-evaluation is a symptom of major depression, a central feature of the cognitive model of depression and is highly characteristic of depression in young people. There is a well-established relationship between low self-esteem (i.e., a more negative self-evaluation) and depression, and low self-esteem predicts future depression symptoms [13, 14]. However, few treatments, including Cognitive Behaviour Therapy [15] explicitly address this symptom. Qualitative research with depressed adolescents suggests that they identify negative self-evaluation as a “big part of depression” and that it is not targeted enough in treatment [14]. Thus, based on feedback from young people and results of previous studies, it is important to better understand the nature of self-evaluation in adolescents and its relationship with depression. This understanding may then be used to adapt psychological models and treatments to the specific experiences and needs of adolescents who have depression.

Self-evaluation can be measured in different ways, focusing on different aspects of the construct. Self-esteem is typically measured using the well-established self-report Rosenberg Self Esteem scale [16]. The RSE is quick and standardised and provides a global assessment of self-esteem on a continuum from positive to negative. Self-evaluation can also be examined as an information processing bias. Self-referential processing refers to the process of how we use perceptions of our self, to guide the evaluation and interpretation of new information [17]. Thus ‘negative self-referential processing’ refers to the tendency to easily associate negative perceptions about oneself (e.g., “annoying”) and difficulties associating positive evaluations (e.g., “smart”; [18]. This is a well examined cognitive bias and studies have shown that compared to participants without a history of depression, formerly depressed patients show negatively biased self-referential processing when in a negative mood state [19, 20]. Similarly, studies have shown that following a negative mood induction, adolescents recall significantly more negative self-descriptions than after a neutral mood induction [21]. Thus this bias appears to be easily triggered (i.e. by a brief mood induction) and long lasting (i.e. in people with a history of depression). Individuals who have negative self-referent processing tend also to also have other information processing biases, including attention and memory biases [19–21]). This co-occurrence of different information processes biases may also predict the onset of future depression episodes and thus constitute a potentially modifiable vulnerability factor [23].

Studies examining negative self-referential processing typically use the Self Referential Encoding Task (SRET) task [22, 23]. In this task participants are presented with a series of adjectives and are asked to rate the words for whether they are self-descriptive. Next, they are given a surprise recall task and asked to recall as many of the adjectives as they can. Using this task, depressed adolescents showed a negative self-referential bias. However, they also endorsed and remembered positive pro-social self-referential words at the same rate as non-depressed adolescents, suggesting that important aspects of positive self-evaluation may remain intact during depression [24]. However, research has identified a specific limitation of using the SRET task with adolescents. A significant number of participants did not recognise or understand some of the words included in the SRET, i.e. ‘feeble’ and ‘pitiful’. This highlights the need to use stimuli that are designed for adolescents and reflect changes in how language is used over time. An alternative strategy to assess self-evaluation in adolescents would be to use adjectives that they themselves have generated to ensure these self-evaluations are familiar.

Anderson (1966) produced a set data norms for 555 descriptive words that have been used to study a range of psychological phenomena including gender stereotypes [25], human attribution processes (self/other judgmental tasks; [26] attachment styles and communication [27], depression [28–30] and self-evaluation in adolescents. For example, one study used trait-words selected from Anderson’s data norms to examine the development of self-evaluation and the impact of social comparison on the valence of self-evaluation across adolescents aged 9–25 years [31]. Other studies have also used trait adjectives from Anderson’s date norms to examine self-appraisal in young adults and healthy adolescents [32]. Anderson’s normative data is clearly flexible and widely used. However, as data were derived from adults and not from adolescents, there is a clear need to create an adolescent specific set of norms that is based on words generated by and used by adolescents rather than adults. Making this resource open-access is important as this could then be used alongside and compliment Anderson’s word-lists. One way in which self-evaluations could be collated is using the Twenty Statements Test (TST; 25), this measure invites participants to generate their own adjectives to describe themselves, in response to a standard prompt, “ I am …….”

Only one study has used an open-response measure to elicit adolescent self-evaluation and measure associations with depression [33]. In this study, the TST was used, however authors coded statements as either ‘positive’, ‘negative’ or ‘neutral.’ A single score was then derived to reflect the overall valence of self-evaluation. Thus, linguistic properties of adolescent self-evaluation which may reflect important nuances in respect to depression remain unexplored. Thus, it may be useful to apply computer linguistic analysis such as Linguistic Inquiry and Word Count (LIWC; [34] as this program codes words into pre-defined categories i.e., ‘positive emotion’ and ‘negative emotion’ and includes sub-categories such as ‘anxiety’ and ‘anger’. The application of LIWC in depression research has demonstrated potential in reflecting the biases typically characterised by depression such as in memory [35] and language [36]. Specifically, research has found that depressed adults tend to recall fewer positive emotion ‘words’ than healthy controls when asked to recall personal experiences (autobiographical memories; [35]. Other literature has also shown increased use of first-person singular pronouns in depressed participants compared to never-depressed controls [37]. Therefore, LIWC may be a useful tool to further explore the specifics of self-evaluation in respect to adolescent depression, given that different components of self-evaluation can be measured separately e.g., positive emotion, negative emotion, anxiety and anger.

The aim of the current study was two-fold; firstly, to examine the specific content of self-evaluation and present a bank of self-referential words generated by healthy adolescents and those with elevated symptoms of depression. This bank of data could be used alongside and complement Anderson’s existing data norms. Secondly, to use linguistic analysis (LIWC) to test the hypothesis that when describing themselves, adolescents with elevated symptoms of depression will use more negative emotion, anxiety, anger and sadness related words and fewer positive emotion words than healthy adolescents.

## Methods

### Participants

Adolescents (n = 1688) from three publicly funded secondary schools in the UK were invited to take part in this study. 919 (54%) provided consent and complete data was provided by 822 young people aged 13 to 18 years (54.7% female; 85.2% White British). From this group we included data from adolescents with ‘elevated symptoms of depression’ i.e., who scored above the clinical cut off for depression (i.e., a score of 27 or above on the Mood and Feelings Questionnaire, MFQ;[38], and ‘healthy adolescents’ i.e., those who scored within the ‘healthy’ range (i.e., a score of 12 or below on the MFQ). The final sample was 549 adolescents; 371 healthy adolescents (58.2% males) with a mean age of 14.85 years (SD = 1.52), and 178 adolescents with elevated symptoms of depression (27.5% males) with a mean age of 14.82 years (SD = 1.29).

### Procedure

This study was approved by the University of Reading Ethics Committee (16/44) and University of Bath Ethics Committee (21–041). Initially, headteachers were contacted and provided information about the study. Following approval from headteachers, information sheets were distributed to all young people aged 13–18 years and to their parents. Young people aged under 16 provided written assent. Parents of adolescents under the age of 16 provided consent via an opt-out method. They were asked to return written forms to the school or contact the researcher (EH) via phone, text or email. All adolescents aged 16 or over provided informed consent.

Adolescents (N = approximately 30 per class) completed paper questionnaires in class in the presence of the researcher (EH). The MFQ was completed first, followed by the TST. Adolescents who did not want to take part were given an alternative activity. All adolescents who took part were entered into a prize draw in which 10 young people from each school won £10 amazon voucher.

### Materials

The Mood and Feelings Questionnaire (MFQ; [38] was used to assess symptoms of depression. This is a 33 item self-report questionnaire. Each statement is rated on a 3-point Likert scale from 0 (not true) to 2 (true). A score of 27 or above is used to indicate an adolescent ‘at risk’ of depression and a score of 12 or below is used to identify adolescents within the ‘healthy range,’ [11, 39]. The MFQ has good reliability and moderate diagnostic accuracy for adolescents [39, 40].

Self-evaluation was examined using the Twenty Statements Test (TST;[41]. This measure provides 20 unfinished sentence stems each beginning with “I am…”. Respondents are asked to define themselves by completing as many statements as possible. Adolescents were not given any example responses and were advised not to think too much about their answers or worry about the order of their responses. Instead, they were advised to write down their answers as they thought of them. Adolescents were advised that they could include any way of defining themselves that they believed was important.

### Data coding

#### Preliminary coding

To create a final dataset which was used in all analyses; only ‘adjectives’ were included. Other statements which included roles (e.g., “a footballer”), physical appearance (e.g.,“blonde”), roles (e.g., “good at football”) were removed. Thus, data from 39 adolescents (N = 312 statements) were excluded from all analyses as they did not generate any adjectives. These data were removed as they either described factual demographic information i.e. “blonde” or described ‘roles’ as defined within research by Rathbone & Moulin (2017) and Hards et al., (2019). Removing these data is consistent with other published research in this area. For example, in a recent paper (e.g. Hards et al.,2020), we used the TST to examine associations between self-evaluation and depression symptoms and excluded any statements which referred to ‘roles’ or statements with no associated valance e.g., “blonde”. Other measures of self-evaluation for example, the Self Referential Encoding Task (SRET) and the Self-Description Questionnaire have also only used adjectives within their items.

Then frequency counts and percentages were calculated for how often each word was generated by adolescents. These words were then organised according to frequency and presented in the open-access resource. To address the first research question, the most common self-evaluation words were then compared between healthy adolescents and those with elevated symptoms of depression.

#### Linguistic coding

Linguistic Inquiry and Word Count (LIWC; [34], a software which conducts text analysis was then applied to the final dataset to examine the second research question i.e. are there any differences in self-evaluation between healthy adolescent and those with elevated symptoms of depression. This programme analyses text and each word is matched against a dictionary with over 6,400 words, producing the percentage (frequency) of words across 90 categories. These 90 language categories have been used to examine a range of psychological processes across many studies such as social relationships, emotionality/affect and cognitive mechanisms [36]. Specifically, ‘positive emotion’ and ‘negative emotion’ categories have been used to measure depressed affect [36]. Cognitive, affective and social categories have also been used to analyse future narrative in adolescents with and without complex pain syndrome [42].

Numerous studies have verified LIWC categories as valid and reliable, with high content and construct validity, superior to other text analysis programmes for identifying ‘emotional expression’ [43]. Specifically, for example, the category ‘positive emotion’ has been found to be reliably reflect positive events and the ‘negative emotions’ category accurately reflects negative events [44]. In this study to examine positive and negative self-evaluation the affect processes dimension was selected. This was broken down into the sub-categories i.e., positive emotion, negative emotion. The negative emotion sub-category was then broken down further into anxiety, anger and sadness.

**Table 1:** The LIWC dimensions and subcategories used in this study with example words

LIWC dimension	Example
**Affect processes**	
Positive emotion	Nice, Sweet
Negative emotion	Hurt, Nasty
Anxiety	Worried, Fearful
Anger	Hate, Annoyed
Sadness	Crying, Sad

## Results

### Preliminary analyses

The most common words generated by participants from each of the three schools were very similar across each of the three schools. Thus, we combined all data and results are reported using this final dataset.

### Word-list: freely accessible resource

Words generated by adolescents are publicly accessible and available here: 10.15125/BATH-01234. There are two data extracts presented in this resource. Firstly, a total 133 words are presented alongside a percentage of how often each word was generated by adolescents. Only words generated by more than 2% of adolescents are included in the first data extract. Secondly, all single words (or very short responses i.e., < 3 words) used by adolescents are presented (N = 402) and a difference score for each word was calculated. This difference score was computed (e.g., % of adolescents in the elevated group who generated word A - the % of adolescents in healthy group who generated word A) so it is possible to identify words more ‘discriminate’ i.e., more commonly used, by those within each depression ‘group’; healthy adolescents vs. those with elevated symptoms. Secondly, to identify words and concepts that were more often used by healthy adolescents and those with elevated symptoms of depression a difference score for each word was calculated. A difference score of a positive value highlights that these words were generated more often by adolescents with elevated symptoms of depression. As shown in the data extract in Table 2, most of these words are negatively valenced (e.g., ‘Sad’, ‘Stressed’). A negative value in Table 2 identifies words generated more often by healthy young people. These words are all positively valenced (e.g., ‘Happy’).

**Table 2:** Words generated by adolescents with difference scores

Word	Difference score
Tired	15%
Sad	13%
Stressed	8%
Useless	7%
Annoying	7%
Shy	6%
Boring	6%
Worthless	6%
Stupid	5%
Weird	5%
Quiet	4%
Confused	4%
Okay	4%
Worried	4%
Scared	4%
Forgetful	4%
Unconfident	4%
Fun	-4%
Smart	-4%
Healthy	-5%
Nice	-5%
Respectful	-5%
Confident	-5%
Helpful	-6%
Cheerful	-6%
Kind	-9%
Funny	-11%
Friendly	-13%
Sporty	-14%
Happy	-28%

### Uses of the word-list

As described these data are freely accessible to researchers who may have interests in using this as a resource to develop adolescent-appropriate research stimuli and modifying existing measures to ensure the words used are appropriate for this population. For example, a frequently used measure of self-referent cognition in depression the Self-Referent Encoding Task (SRET;[45] provides individuals with a list of positive and negative adjectives for people to endorse as self-descriptive. However, using data generated in this study we found that 8/26 positive words (31%) and 17/26 negative words (65%) used in the SRET were either not used by adolescents at all or were generated by fewer than 1% of young people. In relation to Anderson’s word list only 167/555 (30%) of the words spontaneously generated by adolescents in the current study were also present in Anderson’s list.

### Frequency count analysis: most common words generated by adolescents

This study aimed to compare the most common words generated by healthy adolescents and those with elevated symptoms of depression to examine specifics of self-evaluation. Chi-square analysis was computed on the percentage of adolescents who generated each word; with Bonferroni correction applied, adjusted p-value was p = .003.

Table 3 shows the most frequently generated words for both healthy adolescents and those with elevated depression symptoms. Adolescents with elevated symptoms of depression generated negative and positive self-evaluation; two of the most commonly generated words were negative (i.e., ‘Sad’ and ‘Tired’), three were positive (i.e., ‘Kind’, ‘Caring’ and ‘Happy’) and two were neutral (e.g., ‘Shy’ and ‘Sporty’).

**Table 3:** Most common words generated by healthy adolescents and those with elevated symptoms of depression

	Most common words	Elevated Group %	Healthy Group %	χ^2^
	**Elevated Group**			
1	Tired	20%	5%	X(1) = 29.61, p < .001*
2	Funny	16%	27%	X(1) = 8.37, p = .004*
3	Sad	14%	1%	X(1) = 38.62, p < .001*
4	Happy	13%	41%	X(1) = 38.90, p < .001*
5	Shy	12%	6%	X(1) = 5.87, p = .02
6	Caring	11%	11%	X(1) = 0.01 p = .92
7	Kind	11%	20%	X(1) = 6.20, p = .01
	**Healthy Group**			
1	Happy			
2	Funny			
3	Kind			
4	Friendly	6%	19%	X(1) = 15.56, p < .001*
5	Sporty	5%	18%	X(1) = 16.13, p < .001*
6	Caring			
7	Confident	4%	9%	X(1) = 5.36, p = .02

*Note*. Bonferroni correction applied, p < .007

Adolescents with elevated symptoms of depression generated positive prosocial words (i.e., ‘Kind’ and ‘Caring’) as frequently as healthy adolescents. This group described themselves as ‘Tired’ and ‘Sad’ more often than healthy adolescents and there was no significant differences between depression groups in respect to the words ‘Shy’, ‘Confident’. Healthy adolescents only generated positive words i.e. no negative or neutral words. Three of the positive words were pro-social i.e., related to their interactions with others (e.g., ‘Kind’, ‘Caring’ and ‘Friendly’). Healthy adolescents used the words ‘Funny’, ‘Happy’ and ‘Sporty’ significantly more often than adolescents with elevated symptoms of depression.

### Linguistic analysis (LIWC)

We hypothesized that adolescents with elevated depression symptoms would use more negative emotion, anxiety, anger and sadness related words and fewer positive emotion words compared to healthy adolescents. A one-way, between groups MANCOVA was conducted with depression group (healthy or elevated symptoms) as the independent variable and LIWC self-evaluation scores (total affect, positive emotion, negative emotions, anxiety, anger and sadness) as the dependent variables. Results of evaluation of assumptions indicated that normality was not met, however given that MANCOVA is robust to violations of normality, bootstrapping was used [46]. Homogeneity of variance-covariance matrices was not met (Box M = 943.73, p < .001) therefore Pillai’s Trace was used as it is robust to this violation [46]. All other assumptions were met. Age was not associated with depression scores (r_s_ = − 0.01, p = .89, BCa 95% CI-0.07, 0.01], but females (M = 20.88, SD = 16.35) had significantly higher depression scores than males (M = 11.27, SD = 11.77; t(489.11) = 7.68, p < .001; BCa 95% CI [7.16, 12.07], d = 0.3) therefore gender was entered into the MANCOVA as a covariate.

Using Pillai’s Trace, after controlling for gender, there was a significant difference between healthy adolescents and those with elevated symptoms of depression on self-evaluation scores, specifically, the frequency of words classified as negative emotion, positive emotion, anxiety, anger and sadness (V = 0.28, F(6, 502) = 32.09, p < .001, η^2^ = 0.30). Gender was significantly associated with depression symptoms (V = 0.03, F(6, 501) = 2.20, p < .04, η^2^ = 0.02). Follow-up tests (see Table 3) revealed that when controlling for gender, adolescents with elevated symptoms of depression described significantly more words classified as negative emotion, anxiety, and sadness. Healthy adolescents generated significantly words classified as positive emotion. There was a large effect of negative emotions and positive emotion, and medium effects of anxiety, anger and sadness.

**Table 4:** Comparisons between healthy adolescents and those with elevated symptoms of depression on LIWC dimensions

	Mean (SD)		*F*	Effect size (Partial η^2^)
	Elevated symptoms	Healthy		
Total Affect	53.76 (27.75)	60.20 (28.39)	F(1, 507) = 7.65	0.02
Negative emotions	27.64 (25.67)	6.52 (12.80)	F(1, 507) = 132.06*	0.21
Positive emotions	49.41 (29.68)	23.30 (24.38)	F(1, 507) = 90.09*	0.15
Anxiety	10.21 (17.78)	1.93 (5.77)	F(1, 507) = 47.21*	0.09
Anger	5.17 (9.88)	1.12 (4.27)	F(1, 507) = 37.31*	0.07
Sadness	6.51 (14.74)	0.66 (4.07)	F(1, 507) = 44.39*	0.08

Bonferroni correction applied p = .008, *p < .001

## Discussion

One aim of the current study was to create and then share a new database of self-evaluation words, generated by adolescents with and without elevated symptoms of depression. We also compared self-evaluation by adolescents with elevated symptoms of depression and adolescents with low symptoms of depression. Our results suggest that many of the stimuli words used in current measures do not reflect vocabularies used by adolescents to describe themselves. For example, 31% of the positive words and 65% of negative words used in the SRET were not used by adolescents or were generated by fewer than 1% of young people in this study. Additionally, only 30% of the words spontaneously generated by adolescents to describe their self-evaluation are included in Anderson’s word list [47]. These differences highlight why it is important to use words familiar and meaningful to adolescents when conducting research and developing research stimuli appropriate for this population.

An additional aim of this study was to examine the content of self-evaluation using LIWC. Consistent with Beck’s cognitive model of depression [10], adolescents with elevated symptoms of depression described themselves using significantly more words classified as negative emotion, this was a large effect. They also used more anxiety and sadness words than non-depressed adolescents and this difference was a medium effect. Healthy adolescents used significantly more positive emotion words to describe themselves – again this was a large difference between the groups. These findings are consistent with other research which has used LIWC to examine the language used among depressed individuals [37]. For example, [48] found a high proportion of negative emotion words when examining online conversations between patients with Major Depressive Disorder. Importantly, our study represents the first time that a linguistic program has been applied to code self-evaluation generated by adolescents and offers important advantages. Namely, LIWC is the gold-standard tool used to quantify psychological content in written language [44] and uses a rigorously tested coding scheme to categorise data. Secondly, by using LIWC categories it is possible to measure depression affect; this is an essential step in understanding the nature of self-evaluation in this population. However, only single words (or very short responses i.e., < 3 words) were analysed in this study. LIWC is commonly used to analyse articles, expressive writing, blogs, novels etc. [34]. Therefore, future research should use methods which elicit more detailed descriptions from participants as further exploration of self-evaluation using LIWC would be beneficial.

This study also found that adolescents with elevated symptoms of depression generated significantly fewer positive words such as ‘Happy’ and ‘Funny” than healthy adolescents. However, despite this, adolescents with elevated symptoms of depression were able to generate some positive self-evaluation; specifically, they generated prosocial words such as ‘Kind’ and ‘Caring’ as often as healthy young people. These findings provide important contributions to our understanding of self-evaluation in young people as they suggest that despite clinically significant symptoms of depression and negative self-evaluation, some aspects of positive self-evaluation were persistent. It may be that prosocial attributes are ‘protected’ as they are highly salient to adolescents as they typically become more oriented towards interpersonal relationships with their peers during this developmental period [49]. Future research could explore this with qualitative methods to examine the content of self-evaluation in more detail.

The results of this study have several important practical and clinical implications. Firstly, using this database, it is possible to identify words more frequently used by adolescents with elevated symptoms of depression and therefore to build a profile of the content of self-evaluation in this population. This is important given that there has been limited investigations about how adolescents with elevated symptoms of depression describe or think about themselves even though negative self-evaluation is a hallmark symptoms of depression [4]. Secondly, the findings suggest that in respect to depression, a self-evaluation which includes more negative and less positive emotion is typical in young people, consistent with theory. Contrary to the cognitive theory of depression [5], negative self-evaluation among those with elevated symptoms of depression did not relate to feelings of ‘‘worthless’, ‘useless’ and ‘failure’ but instead were more frequently feelings of ‘tired’ ‘sad’ and ‘stressed’. This may reflect the availability and salience of specific ideas and words to young people and the current cultural usage of emotional language. Thirdly, given that this study found evidence to support the presence of some ‘protected’ positive self-evaluation in adolescents with elevated depression, this prosocial self-evaluation may be a useful focus of treatment for depression. Specifically, CBT designed to target low self-esteem and negative self-evaluation has the aim of reducing negative beliefs and replacing these with more positive alternatives [50]. Therefore, it may be the case that with adolescents, prosocial self-evaluation is likely to be a helpful foundation. For example, positive, prosocial self-evaluation may act as building block in therapy and may be a foundation to improve self-evaluation overall. For example, ‘I am a kind person’ could translate and become ‘I am a good person’ [24]. In line with this, given that self-evaluation is likely to change during treatment, it may be important to assess this and monitor it during treatment. Using the adolescent data presented here, it would be possible to construct a self-evaluation measure using vocabularies and descriptions generated by young people themselves.

## Conclusion

This study presents a database of words generated by healthy adolescents and those with elevated symptoms of depression to describe their self-evaluation. These data are freely available for researchers to use to construct or modify measures of self-evaluation or other research stimuli. The findings of this research may have important implications for understanding self-evaluation, a common symptom of depression in adolescents. They suggest that prosocial positive self-evaluation is likely to remain positive, in those with elevated symptoms of depression, despite pronounced negative self-evaluation. Replication with clinical samples and qualitative methods are needed to explore this further to understand the implications on assessment and treatment of depression in adolescents.

## Data Availability

The datasets generated and analysed during the current study are available in the University of Bath repository, 10.15125/BATH-01234.

## Abbreviations

MFQ: Mood and Feelings Questionnaire
TST: The Twenty Statements Test
